# Magnetotelluric evidence for the multi-microcontinental composition of eastern South China and its tectonic evolution

**DOI:** 10.1038/s41598-020-69777-3

**Published:** 2020-08-04

**Authors:** Zhang Kun, Lü Qingtian, Jinhua Zhao, Jiayong Yan, Hao Hu, Fan Luo, Guangming Fu, Xin Tao

**Affiliations:** 1 China Deep Exploration Center-SinoProbe Center, Chinese Academy of Geological Sciences, Beijing, 100037 China; 2 China University of Geosciences (Wuhan), Wuhan, 430074 China; 3 East China Institute of Technology, Nanchang, 330013 China

**Keywords:** Geodynamics, Geophysics

## Abstract

The tectonic boundaries and geodynamic evolution of the South China Block are widely debated. While the community largely agrees on the occurrence of the collision between the Yangtze and Cathaysia Blocks, the lack of ultrahigh-pressure metamorphic rocks and obscurity of the boundary lead to inconsistencies among the abundant geological and geophysical data. We present three profiles that reveal the geoelectrical structure of eastern South China. Distinct conductive interfaces oriented NE–SW are identified in the geoelectrical lithosphere, which separate the region into six parts. To interpret our observations and resultant model, we develop and propose a mechanism of “microcontinent interaction”. Our new model justifies the prior proposed models of ‘block collision’ and additionally proposes ‘multi-terrane accretion-collision’ to address the tectonic evolution.

## Introduction

**Figure 1:**
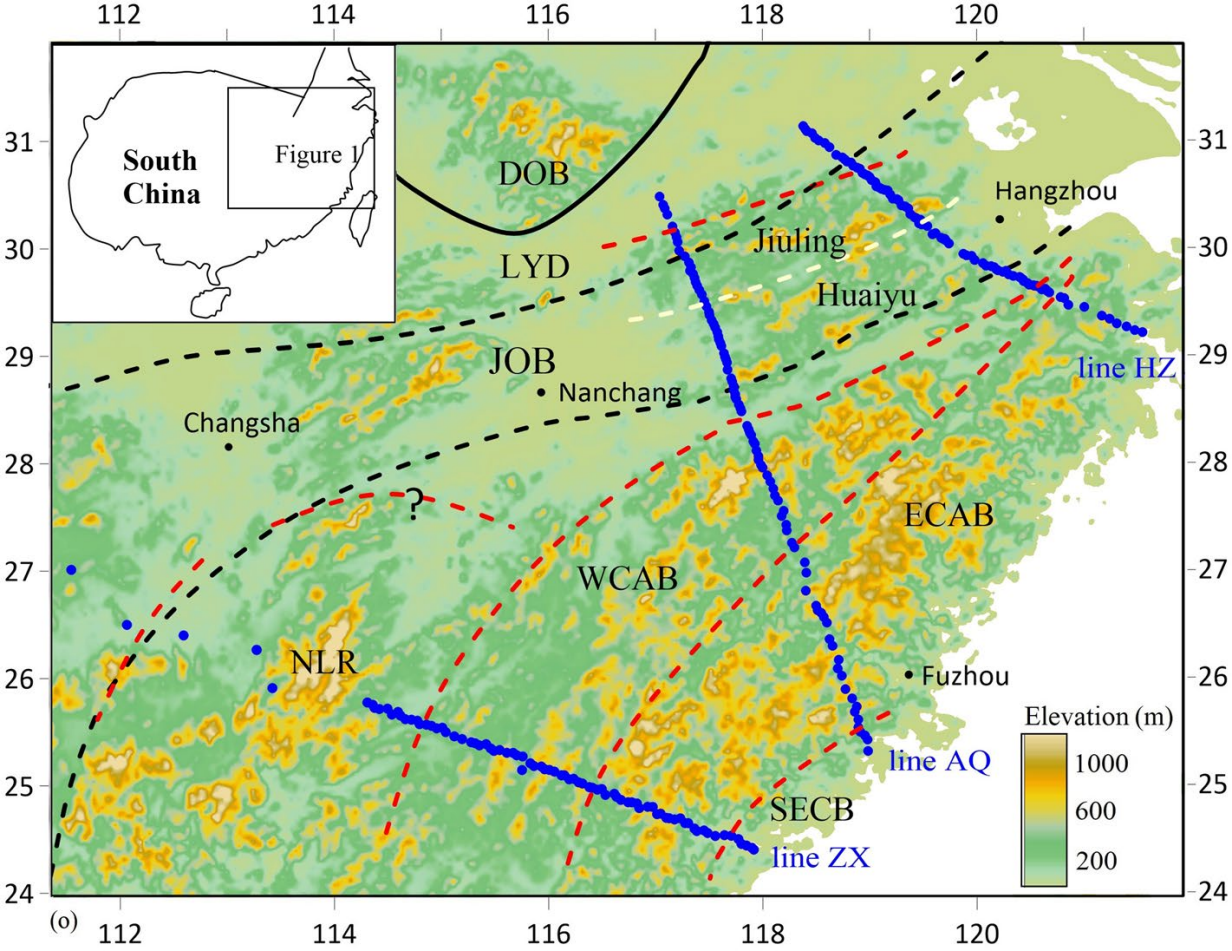
Topographic map with magnetotelluric stations and tectonic boundaries (the map is plotted with a commercial program named Encom PA V12.0, https://www.pitneybowes.com/us). MT stations are shown by blue dots; previously proposed tectonic boundaries are shown by black (dashed) lines^51^; proposed deep tectonic boundaries in this study are shown by red dashed lines; the terrain boundary is shown by a yellow dashed line. *DOB* Dabie orogenic belt, *LYD* Lower Yangtze depression, *JNO* Jiangnan orogenic belt, *WCAB* West Cathaysia Block, *ECAB* East Cathaysia Block, *SECB* southeast coast belt, *NLR* Nanling Range, *WYR* Wuyi Range in the western WCAB.

With the closure of the South China Ocean, South China was formed from the Neoproterozoic collision of the Cathaysia and Yangtze Blocks^1^, where the Jiangnan orogenic belt marks the suture^2^, and was generally accepted to have collided with North China from the Indo-Sinian to the early Yanshanian (Mesozoic), forming the Dabie orogenic belt and the Lower Yangtze depression on the northern margin of eastern South China (e.g.,^3,4^) (Fig. 1). However, Yangtze and Cathaysia have mainly been regarded as two unified geological units in most geodynamic studies. This thesis led to wide debates on the tectonic boundaries and processes of the continent (e.g.,^3,5–7^). Recent geological evidence, such as the discovery of distinct multistage magmatic and metamorphic events in the western and eastern parts of Cathaysia, tends to favor multiple terranes involved in South China and especially indicates that Cathaysia was a result of accretion and collision between West Cathaysia and East Cathaysia^8^, which used to be separated. In addition, several extensive crustal/lithospheric thinning subregions revealed by the velocity model from a seismic study indicate the nonuniformity of the lithosphere^9^. This contradiction suggests that the interactions of microcontinents and the geodynamic processes of intracontinental tectonic evolution were more complex than previously predicted and may not be explained by a single simple mechanism, which implies the need for robust and higher resolution constraints on the physics of the lithosphere.

**Figure 2:**
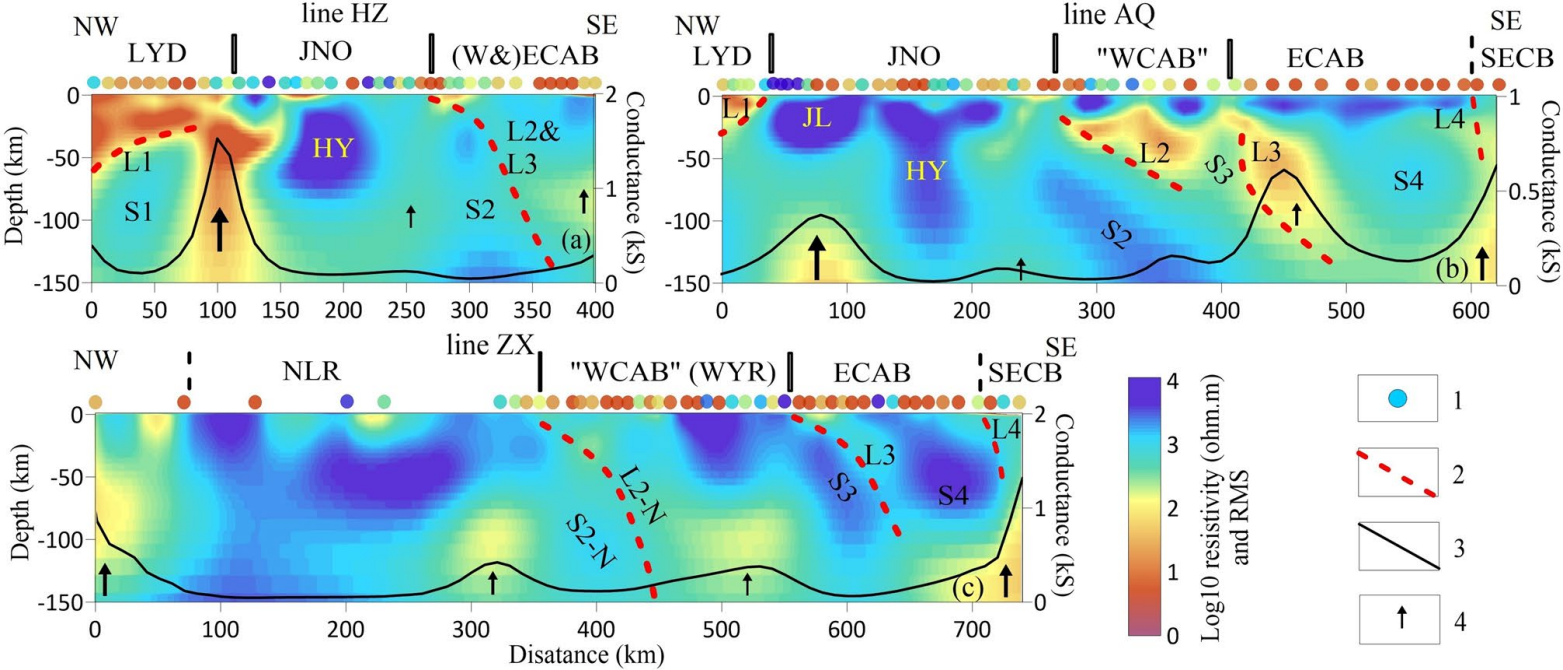
2D inversion models of line HZ (**a**) with normalized misfit 2.265, line AQ (**b**) with normalized misfit 2.379 and line ZX (**c**) with normalized misfit 1.91. 1: normalized misfit of the inversion at each station using the same color bar with electrical modes; 2: proposed slab interfaces; 3: depth-integrated conductance calculated for the upper mantle (50–150 km); 4: upwelling direction. S1–S4 represent the “traces” of intracontinental subducted slabs, L1–L4 represent the top interfaces of the slabs, and “N” (S2-N, L2-N) indicates the Nanling Range.

The magnetotelluric method observes natural electromagnetic signals on the surface to probe the subsurface conductivity^10^. It has been widely used to constrain the structure and status of the crust and mantle (e.g.,^11^) because of its sensitivity to fluids and melt phases. As a part of the National Key R&D Program of China, data from 249 magnetotelluric stations (Fig. 1) were acquired along three profiles. Our new resistivity model suggests that South China contains at least the lower Yangtze depression, the Jiangnan orogeny (Nanling Range in the southwest), West Cathaysia and East Cathaysia (Fig. 2a–c). In addition, the Southeast coast belt might have been located on the margin of an independent continent towards the far east of East Cathaysia (Fig. 2b,c).

## Magnetotelluric inversion and interpretation

After data processing, we analyzed and inverted the sorted data in the period range of 0.01–1,000 s (see “Methods”). The 2D preferred models constrain the electrical structure from the upper crust to the upper mantle at a depth of 150 km (Fig. 2). Furthermore, model tests for structures interpreted in the results and discussions have been carried out (see “Methods”). In our models, we identified four conductive layers (L1–L4 in Fig. 2, < 200 Ω m) in eastern South China. The conductors detected in magnetotelluric results are due to many factors, such as fluids, metasediments, and iron oxides. Conversely, the category of an interconnected conducting mechanism can be excluded due to the presence of detected resistors. Previous magnetotelluric studies suggested a few deep weak layers characterized by low resistivity, which are probably generated from relatively high fluid contents (e.g.,^12^). In addition, weak crustal layers and lithosphere interfaces between different blocks are revealed by velocity models from receiver functions and surface wave studies^9^. The “crocodile” structure beneath the Lower Yangtze and several deep interfaces are revealed by the results of deep seismic^13,14^ and magnetotelluric studies^4^.

Many studies have revealed that passive continental subduction might follow oceanic subduction in a process of continent–continent collision^15^. The possible pull exerted by the subducted oceanic slab can be the cause of this continental subduction, which is supported by 3D numerical models^16^. Furthermore, during the subduction process, a weak layer above the subducted slab can be created by the infiltration of fluids after dehydration^17^. In our geoelectrical models, the resistors (S1–S4, 1,000–10,000 Ω m) beneath L1–L4 have similar characteristics of slope, large distribution range and deep vertical extent. Considering the residual electrical structures of subduction–collision between the Yangtze, Cathaysia and North China Blocks, four layers (L1–L4) are inferred as weak layers (detachment layers), which indicate possible subduction–collision processes. They can also be used to distinguish six distinct regions in our study area with the corresponding intracontinental subducted slabs, which are represented by the “traces” (S1–S4) revealed in our models.

### Lower Yangtze depression

The Lower Yangtze depression contains a relatively high-resistivity upper crust (~ 0 to 10 km, > 100 Ω m) and a low-resistivity detachment layer with depths ranging from the upper crust to the upper mantle, which overlie L1 (~ 10 to 30 Ω m, Fig. 2a,b). Underlying the detachment layer, a thick resistive layer (> 2000 Ω m) extends westward from the lower crust to the upper mantle with a total top dip of 22° (calculated from L1). Under the western adjacent profiles, a structure resembling the shape of a “crocodile” beneath the middle to lower reaches of the Yangtze River and the Dabie orogen was proposed earlier by seismic reflection waveforms and resistivity models^4,13,14^. This structure is believed to be a “trace” of intracontinental subduction after continental collision, which is revealed by ultrahigh-pressure metamorphic rocks^18^ in Dabie. There is a cold lithospheric mantle beneath the Lower Yangtze depression, supported by low geothermal heat flow values (data downloaded from https://chfdb.xyz/show.html). Therefore, a subducted remnant can be inferred and is characterized by this thick high-resistivity layer (beneath L1, 400–2,000 Ω m) as the intro-continental subducted slab (S1). Moreover, the vertical conductive belt and depth-integrated conductance southeast of L1 (profiles 80–120 km in line HZ and 50–100 km in line AQ, > 400 S) indicate that this slab has broken off and that a channel has been formed for the upwelling of deep material during evolution.

### Jiangnan Orogen and Nanling range

The Jiangnan orogen consists of three resistors (> 4,000 Ω m) in the crust and upper mantle, intruded and separated by an underlying widespread conductor and a vertical conductive belt (Fig. 2a,b, < 150 Ω m). The western and middle resistors with different thicknesses can represent the Huaiyu and Jiuling terranes. They are characterized by a thick sequence of different Precambrian basement rocks^8^ separated by a fault. The electrical structure of the Jiuling and Huaiyu terranes shows a possible connection between the western (S1) and eastern (S2) Yangtze subducting slabs, respectively. The lithospheric thickness in Huaiyu is thicker than that in Jiuling, and the lithosphere becomes thinner to the northeast (Fig. 2a,b). The distributions and vertical extents of these two terranes are consistent with the seismological Moho depth^19^ and the crust-mantle destiny difference estimated from gravity data.

The discovered surface mafic magmatism (830–820 Ma^20^, peraluminous (S-type) granites (835–820 Ma^6^ and magmatite (ophiolite) belt^7^ support the interpretation that subduction-collision event occurred along the Jiangshao fault^1,6^. This is also supported by the observed high gradient of geothermal heat flow and the seismological Moho depth. The remnants of an intracontinental subducted slab (S2) revealed in our models show the result of (de)coupling between the Yangtze and Cathaysia Blocks. More details show a “C”-shaped contact boundary composed of the remnants and their overlying conductive structures. This structure could reconcile prior geological and geophysical data for the much debated subduction-collision direction. It is pertinent to infer that this mechanism is westward subduction based on shallower studies of the upper to middle crust of the study region. The overlying crustal conductive belt is likely to represent a postcollisional extension system. This is also supported by the observations of igneous rocks (850–750 Ma^21^, mafic dikes (800–760 Ma^22^, and multistage magmatism since the Paleozoic^23^. However, the electrical structures in our models indicate an opposite subduction mechanism, showing the wedging of the Cathaysia lithosphere into the lower crust of the Yangtze Block.

The electrical structure of the Nanling Range (profile 80–370 km in line ZX, Fig. 2c) includes shape, large scope and deep extent. It is distinctly different from those of Jiangnan and Cathaysia. Moreover, the structural difference is in accordance with the distinct distribution of the seismological Moho depth and crust-mantle density difference. The local resistor (S2-N, ~ 2000 Ω m) in the upper mantle west of L2-N has characteristics similar to those of the east Yangtze slab (S2) along line HZ. Unlike the electrical structure of the two terranes, our model reveals a relatively stable geoelectrical lithosphere and another remnant of an intracontinental slab and extends eastward from the lower crust. This is similar in many ways to the characteristics of S1, such as the large-scale layered structure. The southeastward dip of S2-N (~ 60°) is much larger than that of S2 (~ 42°). However, the breakoff level of S2-N is shallower. This is revealed from the stronger continuity with the western resistor. However, the larger depth-integrated conductance (profiles 280–330 km and 480–540 km in line ZX, ~ 400 S) shows stronger action of deep material on both sides of S2-N. These features indicate the shorter subduction time but stronger underplating and upwelling of hot material beneath the Nanling Range than beneath Jiangnan. Moreover, the geoelectrical lithosphere of the Nanling Range, probably characterized by a craton, is revealed as a widespread resistor (> 2000 Ω·m) at depths greater than 150 km, in contrast to Jiuliang and Huaiyu. Unfortunately, the western boundary cannot be resolved precisely due to the large station distance.

### West Cathaysia

West Cathaysia consists of a resistive crust (> 2000 Ω·m) intruded and separated by a detachment layer (< 200 Ω·m) overlying the subducted slab (S2 and S2-N). A varying thickness of the geoelectrical lithosphere is shown in the NE direction, but there is no major lateral variation. Similar to the structure of S2, a remnant of an intracontinental slab (S3, 300–3,000 Ω m) is revealed beneath L3 (Fig. 2). Its extension eastward from the middle crust shows an increase in resistivity and thickness to the southwest. The structure of S3 and its overlying conductor indicates an evolution similar to that of the eastern boundary of the Yangtze Block.

The Neoproterozoic magmatites (ophiolite and gabbro^7^, indicate a few interactions between West and East Cathaysia^24,25^). These are inferred to be distinct regions based on the horizontal discontinuity of the electrical structures in our models. Further evidence comes from gravity anomalies^26^, seismicity distribution (data from https://www.csndmc.ac.cn), seismological Moho depths, and geothermal heat flow differences. Furthermore, the thin geoelectrical lithosphere suggests that there is not a cratonic basement under West Cathaysia, which might have acted as a buffer in the collision between the Yangtze and Cathaysia Blocks and prevented the penetration of the ultrahigh-pressure metamorphic rocks into Jiangnan^5,6^. The vertical conductive belt and depth-integrated conductance (east of L3) are larger in the northern part of West Cathaysia (profile 420–480 km in line AQ). The weaker extension of the lithosphere of southern West Cathaysia (profile 600–630 km in line ZX) indicates stronger compression during the continued evolution after the collage with a block on the east. This compression might have been related to the movement of the Nanling Range.

### East Cathaysia and Southeast Coast Belt

To the west of a Cretaceous magmatic belt, East Cathaysia consists of a large resistor (> 2000 Ω m) extending from the surface to the upper mantle with a mid-crustal conductive layer (< 200 Ω·m) intruded into the resistor (Fig. 2b,c). Our models show little or no major lateral variations at depth, except for the presence of a resistor. The models also indicate a strong influence of the upper mantle on the eastern part of the study region. Here, the bulk composition of the lithosphere might be the cratonic part of the whole of Cathaysia. This result is suggested from the interpretation of the residual melt from the Paleoproterozoic granitic basement^27^. However, the distinct geoelectrical structure (widespread conductive anomaly and large depth-integrated conductance) and stress pattern^28^ in the Southeast coast belt suggest a tectonic boundary (profiles 600 km in line AQ and 710 km in line ZX). This boundary is also supported by the geothermal heat flow value and the seismological LAB depth^29^. The deep conductor can be interpreted as the representation of the upwelling heat flow and spreading of the hot material. We propose that the Southeast coast belt is a contact zone, which broadly coincides with the distribution of the major Cretaceous mafic magmas^30^.

## Discussion

### Assembly process of eastern South China

**Figure 3:**
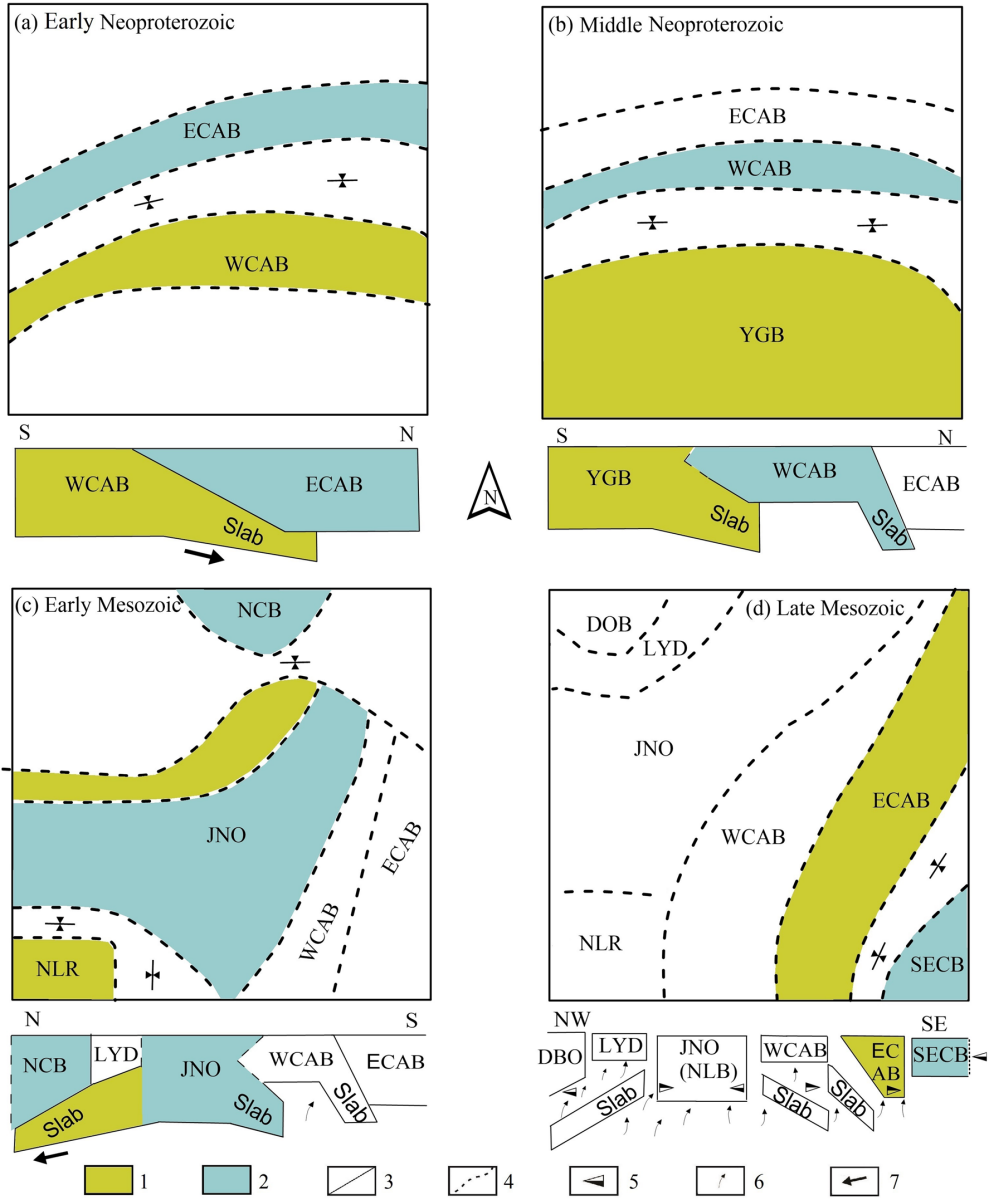
Tectonic processes in the eastern SCB. All of the microblocks here are modified to the coincident direction and relative position for each stage of this process, based on the paleomagnetic data (www.scotese.com^52^). (**a**) Accretion between the ECAB and WCAB in the middle-late Neoproterozoic; (**b**) collision between the CAB and YGB in the late Neoproterozoic; (**c**) interaction among JNO, NCB and NLR in the early Mesozoic; (**d**) accretion between the SECB and ECAB in the late Mesozoic. 1: lower plate; 2: upper plate; 3: proposed regional boundaries; 4: inferred regional boundaries; 5: stress direction; 6: upwelling direction; 7: subduction (opposite obduction) direction. The WCAB subducted towards the ECAB before the middle Neoproterozoic, and the whole CAB then collided with the YGB. After Paleozoic sedimentation and tectonism, the NLB and JNO subducted northward in the Indosinian, and the suturing of the ECAB and SECB occurred under intracontinental extension of the SCB and compression from the Paleo-Pacific block.

Material flow indicates large-scale tectonic movements that can be driven by the pressure gradients from topographic (downslope) variation (see “Methods”), which has been estimated on the northern margin of the Tibetan Plateau (120 Pa/m)^31^. The pressure gradient (< 39 Pa/m) in our study area is much smaller than that of the active continental margin (Tibet). In addition, geological evidence indicates large-scale tectonic movements after the Mesozoic, which are rarely found along the coupled structural cross profile on the surface^32^. Accordingly, we propose four key stages in the geodynamic evolution to constrain the assembly process of eastern South China (Fig. 3) from the middle Proterozoic to the late Mesozoic.

#### Early Jinningian collision

We propose that Cathaysia formed in the middle-late Proterozoic by the amalgamation of West and East Cathaysia, where a structure similar to that of a “suture” is revealed in resistivity models. Arc basaltic magmatism (978 ± 10 Ma) and OIB-type gabbro (847–818 ± 9 Ma)^7^ are related to this tectonic event, and mafic–ultramafic suites (ca.850–800 Ma) with intra-plate geochemical signatures^33^ indicate the re-activation of the “suture”. These ages are used to constrain the collision time prior to the collision between the Yangtze and Cathaysia Blocks (Fig. 3a).

#### Late Jinningian subduction–collision process

Many pieces of evidence, such as arc (e.g., 878–822 Ma^22^, and mafic magmatism (e.g., 830–820 Ma^7^, are interpreted as indicating subduction-collision processes between the Yangtze and Cathaysia Blocks in the late Proterozoic. However, the mechanisms for the process remain subjects of debate, and many different models have been proposed based on petrochemical studies (e.g.,^6^). The discovered remnants of the intracontinental subducted slab beneath Jiangnan can be used to constrain the structure of the collisional suture, which is indicated as a result of the east-directed subduction–collision process. The wedging of the West Cathaysia lithosphere can thus be inferred from the decoupling between the lithospheric mantle and the crust of the Yangtze Block, where the crustal thrust and lithospheric mantle subducted eastward. The lithospheric mantle thinning towards West Cathaysia can be attributed to partial melts, with coexisting hydrous fluids from slab dehydration. This interpretation is in line with the models of geodynamic evolution after subduction^34^. Our hypothesis (Fig. 3b) can reconcile the cause of homogenous material in the basement rocks of the Yangtze and Cathaysia Blocks and the major conflict between different geodynamic models.

#### Indo-Sinian collision

During the Indo-Sinian orogeny in the Yangtze Block, another geological terrane, referred to here as the Nanling Range, moved towards Jiangnan and West Cathaysia. This terrane is revealed by an E–W-trending Triassic fold system in southern Jiangnan^7^ and the shortened S2-N beneath Cathaysia. The observed data for bimodal granite (239–231 Ma) and biotite granite (239 Ma) present in the Nanling Range were used to calculate the diagnostic Rb/10-Hf-Ta*3 values^35^, indicating a syncollisional characteristic^36^. The geoelectrical structures revealed in the resistivity models indicate a collision event between West Cathaysia and Nanling. This lithosphere of Nanling Range may also have wedged into Jiangnan and developed coevally with the collision between the Yangtze and North China Blocks (Fig. 3c).

Moreover, the lower intercept ages (250–230 Ma) of monzonite gneiss, migmatite, and felsic metavolcanic rocks reveal a metamorphic event along the boundary between West and East Cathaysia^8^. This metamorphism has the same tectonic significance as the Mesozoic overthrust tectonics proposed by Hsü et al.^37^ along the boundary between Yangtze and Cathaysia. These tectonic events indicate that the sutures might have been reactivated by the remote effects of the collision to the west. These microcontinents might have rifted or experienced complex tectonic processes, which might have been caused by the evolution (subduction and breakoff) of previous intracontinental subducted slabs (S2 and S3) from the Neoproterozoic to the early Mesozoic.

#### Yanshanian extension and contact motion

Bimodal volcanic rocks and A-type granites, characterized by younger ages, lie towards the east since the late Mesozoic and are mainly distributed along the suture zones in Cathaysia^26^. The temporal and spatial distributions of the late Mesozoic magmatism indicate an intracontinental extension event. This event was active in the west and developed towards the east. We suggest that the mechanism of this type of extension is due to the breakoff of the subducted slab (S1) between the Yangtze and North China Blocks^4^. A slab breakoff can lead to the upwelling of hot material and interactions with the lithosphere. The sutures in the microcontinent in South China appear to have been reactivated within a distance on the order of magnitude of that from Jiangnan to East Cathaysia. As a result, continental lithosphere and subducted slabs, characterized by high resistivity, are separated by deep conductive belts (Fig. 3d).

Unlike the late Mesozoic igneous rocks, the Cretaceous mafic rocks are mostly distributed in the Southeast Coast belt^38^, yielding ages of 118–107 Ma^26^. Previously observed data for gabbros and I- and A-type granites, yielding an age of 115 Ma, are interpreted as associated rocks^39^. The spatial distribution of Cretaceous magmatism indicates a collisional event between East Cathaysia and the Southeast coast belt that generated a back-arc system. A regional unconformity between the upper and lower volcanic series^40^ indicates compressive stress from northwest to southeast^26^. We propose that the collision was driven by west-directed compression from the Paleo-Pacific Block and east-directed compression from intracontinental extension in South China (Fig. 3d).

### Comparison with evolution of American coastal regions

In the Pacific Rim, our proposed model is different from the construction of the Cordilleran orogen. Here, the Farallon plate subducted beneath a coherent western North American plate margin from the latest Jurassic. The slab flattening and its rollback may have been the cause of local extension in the hinterland^41^. However, the Paleo-Pacific plate, acting through remote influence, may have played an important role in the Middle Jurassic compression of South China. When the Paleo-Pacific plate started west-directed subduction in the Middle Jurassic, the lithospheric mantle of South China had been thinning since the Paleozoic^42^. Conversely, our model is similar in many ways to the evolution of the Appalachian orogen in eastern North America and the formation of Pangaea. Here, the accretion of Ganderia, Avalonia and Carolinia to the eastern margin of Laurentia mainly resulted in the formation of Laurussia, preceding the Carboniferous continent–continent collision with Gondwana^43^.

## Concluding remarks

We conclude that at least six microcontinents are included in eastern South China, and a model with multiple collisions can describe the assembly evolution. West Cathaysia was a microcontinent that contacted East Cathaysia during the early Jinningian. This collisional event was related to the assembly of South China. The process of subduction–collision developed between the Cathaysia and Yangtze Blocks, in which West Cathaysia played a role as a buffer. During the Indo-Sinian, the northward motion of the Nanling Range led to its collision with Jiangnan and West Cathaysia, which was coeval with the collision between the Yangtze and North China blocks, and the eastern sutures were reactivated under the influence of the collision. After the conversion of the tectonic regime from compression to extension in eastern South China, contact between East Cathaysia and the Southeast coast belt occurred. This was the result of their collision, which was driven by the intracontinental extension of South China and west-directed compression from the Paleo-Pacific block. We interpret the tectonic evolution of the South China Sea as a postcollisional process between South China and a “block” to the east during the Cenozoic.

Due to the limitation of data coverage (lack of marine data), our model cannot constrain all the structures east of the coastline. A magnetotelluric design for marine profiles can test our hypothesis for the Southeast coast belt. Furthermore, it should be noted that other findings (e.g.,^8^) also indicate the possibility of more microcontinents and more accretional–collisional events than those presented in the current model, especially during the Paleozoic.

## Methods

### Data acquisition and processing

Data from 249 magnetotelluric stations have been acquired using commercial MT instruments, namly Phoenix MTU-5A. The time series of magnetotelluric (MT) data recorded more than 40 h. It contains two electrical components (Ex and Ey; x is north and y is east) and three magnetic components (Hx, Hy, and Hz). The data are processed using statistically robust algorithm^44^ with remote-reference technique to calculate the transfer functions. Hence, the impedance tensor and tipper are obtained after spectrum editing with a broad period range of 0.01–1,000 s. It is more than sufficient for probing the lithosphere despite conductive region in the lower crust. Although, the apparent resistivity (ρ =|Z|2/ωμ) and the impedance phase (P = atan[Zima/Zreal]) data are generally of high quality, we employ an automatic de-noising^4^ technique wherein data that are not a good fit for the 1D inversion. After removing the noise, we manually select the data to met the quality standards and removed automatic method. Finally, we sort out the data into a unified frequency list with seven points in one decade (Supplementary Figures S1–S3).

### Phase tensor analysis

We use phase tensor analysis^45^ to determine the dimensionality of the underground material from typical MT data. The phase tensor skew angle β, indicates the asymmetry in the MT response and reflects 3D structures, as shown by the color, filling the phase tensor ellipses (the material approaches to 2D whenβ approaches to 0). Please note that Φ (2 × β) was plotted instead of β for better display of the asymmetry of the MT responses^46^. The polar direction is represented by the ellipses’ major and minor axes. The ratio and complexity is used to represent the dimensionality.

At the different depths of crust and upper mantle (Supplementary Figure S4), the orientations of phase tensor ellipses generally show NE–SW direction in the middle study region. The light colors (low values) of β and low ratio of the axes suggests 1D or quasi-2D structure. However, the orientation of the phase tensor ellipses rotates to a dominant EW–NEE direction from the western ZX to the middle HZ, and rotates to a NWW–NW direction in the northern and southern study area. Three major geo-electrical belts are reflected differently in the lower crust/upper mantle from northwest to southeast. A nearly flattened ellipses with high ratio of the axes and β values (dark colors) suggest the existence of abrupt lateral geo-electrical interfaces or parallel or asymmetric conductive structure (3D structure), which mostly indicate the boundaries of micro blocks. Further, a common strike angle cannot be identified easily from the data. But a tendency towards the strike angle is reflected of the order of – 30° N to 75° E (mainly from NW to NEE direction) at all the periods.

### Inversion and tests

We invert our MT data using non-linear conjugate gradients (NLCG) method^47,48^. The objective function and its gradient are expressed as follows:

1 $$\begin{gathered} \varphi = \sum {[(d - F(m))/\varepsilon ]}^{2} + \lambda m^{{\text{T}}} W^{{\text{T}}} Wm \hfill \\ \partial \varphi_{d} /\partial m = - 2{\text{Re}} \sum {((d - F(m))/\varepsilon ) \times \partial F/\partial m} \hfill \\ \end{gathered}$$

where φ is the object function *d* is the apparent resistivity (and impedance phase); *ε* is the data error; *F*(*m*) is the forwarding factor; _*λ*_ is a regularization factor; *m* is the model; and *W* is the model covariance matrix.

The search length and direction are expressed as follows:

2 $$\begin{gathered} p_{0} = - C_{0} (\partial \varphi_{0} /\partial m_{0} ) \hfill \\ p_{k} = - C_{k} (\partial \varphi_{k} /\partial m_{k} ) + \beta_{k} p_{k - 1} ,\quad k = 1,2, \ldots \hfill \\ \beta_{k} = \frac{{(\partial \varphi_{k} /\partial m_{k} )^{{\text{T}}} C_{k} ((\partial \varphi_{k} /\partial m_{k} ) - (\partial \varphi_{k - 1} /\partial m_{k - 1} ))}}{{(\partial \varphi_{k - 1} /\partial m_{k - 1} )^{{\text{T}}} C_{k - 1} (\partial \varphi_{k - 1} /\partial m_{k - 1} )}} \hfill \\ m_{k,l} = m_{k} + \alpha_{k,l} ,\quad l = 0,1,2, \ldots \hfill \\ \alpha_{k,l + 1} = \alpha_{k,l} - \frac{{(\partial \varphi_{k,l} /\partial m_{k,l} )}}{{p_{k}^{T} H_{k,l} p_{k} }} \hfill \\ \end{gathered}$$

where p is the search direction; α is the search length; l is the line search number; H is a quadratic function of the object function; and C is a preconditioning factor. One potential way to define the preconditioning factor is proposed by Zhang et al.^49^:

3 $$\begin{gathered} C_{l} (j,k) = [\upsilon_{l} (j,k)I + \lambda W^{T} W]^{ - 1} \hfill \\ \upsilon (j,k) = \frac{1}{{\sum \varepsilon /(d^{obs} - d)m(j,k)^{2} }} \hfill \\ \end{gathered}$$

where $\upsilon$ is a non-independent coefficient for cell (j,k).

Parallel structures are used to incorporate frequency data for the computation efficiency, and a topography (marine) factor is added for the practicality of the algorithm. Furthermore, the static shift correction method^49^ is used during inversion for high accuracy and 3D electrical structure restoration. The correction method uses the apparent resistivity and impedance phase data to define the static shift and update the initial model. So the distorted data can be fitted well in the inversion, through which the 2D model gives consideration to 3D structures also using the diagonal components.

For the preferred models, the domain is 8,000 km (Y) × 1,200 km (Z) for profile HZ; 12,000 km (Y) × 1,200 km (Z) for the profile AQ; and 20,000 km (Y) × 1,200 km (Z) for profile ZX. The mesh spacing in the Y direction is ~ 3 to 5 km within the profile depending on the stations distribution, and increases progressively with a ratio of 1.5 outside. The mesh thickness (Z direction) starts with several shallow layers of 20 m, is used for static shift correction^49^ and allows inversion generating minor structures to address any local galvanic distortion/topographic effects^11^. The background resistivity of the initial models is 100 Ω m. This is updated by the static shift correction on the surface. The TE, TM, and TP data at 40 periods from 0.01 to 1,000 s are used in the 2D inversion, with an error floor set to 40%, 10% and 0.1, and the regularization factor is 15. The observed and computed data (root mean square error of the data and inversion responses) fit for each station is shown in Fig. 2. The comparison of curves are shown in Supplementary Figures S1–S3.

To identify the best models, we test inversions based on the data error, data type (combination of TE, TM and TP data), amount of data, model setting, background resistivity of the initial model, and regularization factor. In addition, the anomalies at deeper depth are tested by forward modeling and long period data are tested by constrained models (Supplementary Figure S5). It is tempting to attribute the conductive anomalies are due to the existence of south-eastward lower crustal flow. This indicates the weak layer above the slab, if the separated conductors can be connected in the test region. For testing this possibility, a moderate conductive (50 Ω m) channel is introduced to the preferred model. We perform a test inversion using the modified model as the starting model. However, a resistor occurred and the data cannot be justified. The overall misfit of the test inversion is larger than the original inversion (2.41 vs 2.37). This indicates that a high resistivity anomaly exists in the test region, separating the conductors. Accordingly, we infer the high resistivity anomaly is the reflection of a slab, with due consideration of other evidences.

### Pressure gradient

The pressure gradient can be calculated by $\nabla p \approx \left( {\frac{{\rho_{m} - \rho_{c} }}{{\rho_{m} }}} \right)\frac{{\rho_{c} g\Delta h}}{L/2}$^50^, where $\nabla p$ is the pressure gradient, $\Delta h$ is the topographic variation over a distance *L*, $\rho_{c}$ and $\rho_{m}$ are crust and mantle densities. Estimating a crust density of 2,800 kg/m^3^, the greatest lateral pressure gradient can be calculated with the greatest topographic variation (700 m) in a shortest distance of the tectonic units (150 km) and a mantle density of 3,300 kg/m^3^ in our study area.

### Tips

Most of the supporting evidences for our interpretation and discussion are shown in Supplementary Fig. S6 for details, which contain heat-flow, Moho depth, destiny difference of crust and upper mantle, LAB depth, magmatism location.

## Supplementary information

Supplementary information.
