# Supplementary material for: Magnetotelluric evidence for the multi-microcontinental composition of eastern South China and its tectonic evolution
Source: Sci Rep. 2020 Aug 4;10:13105. doi: 10.1038/s41598-020-69777-3 (PMC7403324; doi:10.1038/s41598-020-69777-3)
Supplement: Supplementary file 1 — Supplementary information. [file 41598_2020_69777_MOESM1_ESM.pdf]

Supplementary Information

Magnetotelluric evidence for the multi-microcontinental composition of eastern South China and its tectonic evolution

Zhang Kun[1], Lü Qingtian[*,1], Zhao Jinhua[1], Yan Jiayong[1], Hu Hao[2], Luo Fan[3], Fu Guangming[3], Tao Xin[3]

1 China Deep Exploration Center-SinoProbe Center, Chinese Academy of Geological Sciences, Beijing 100037，China

2 China university of Geosciences (Wuhan), Wuhan, 430074, China

3 East China Institute of Technology, Nanchang, 330013, China.

* Corresponding author: **Lü Qingtian**，E-mail address: lqt@cags.ac.cn.

1. Supplementary Figures

    Figure S1. Comparison of the observed data and inversion responses located on line HZ (a-h), and normalized misfit for each iteration of the inversion (i).

    Figure S2. Comparison of the observed data and inversion responses located on line AQ (a-h), and normalized misfit for each iteration of the inversion (i).

    Figure S3. Comparison of the observed data and inversion responses located on line ZX (a-h), and normalized misfit for each iteration of the inversion (i).

    Figure S2. Skewness of the phase tensor.

    Figure S3. An example of model tests for the inversions.

    Figure S4. Typical evidences for the tectonic evolution of South China.

2. Supplementary References

1. Supplementary Figures

Figure S1. Comparison of the observed data and inversion responses located on line HZ (a-h), and normalized misfit for each iteration of the inversion (i). The preferred model is obtained after 115 iterations, which normalized misfit is 1.89 decreasing from a starting model misfit of 12.26. This indicates that the inversion has found a model that fit the data well.

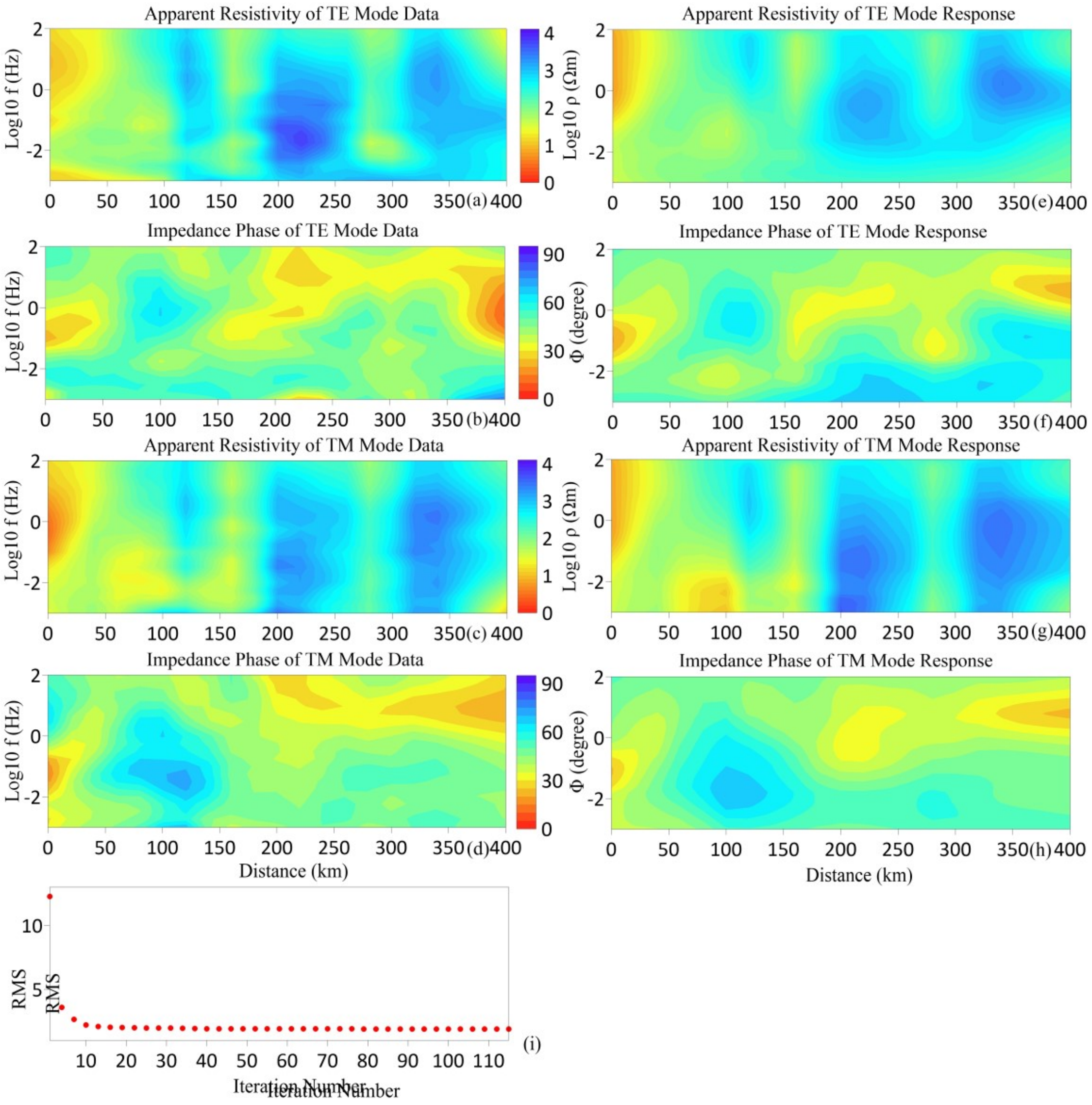

Figure S2. Comparison of the observed data and inversion responses located on line AQ (a-h), and normalized misfit for each iteration of the inversion (i). The preferred model is obtained after 178 iterations and fits data well with normalized misfit of 2.37 (from a starting model misfit of 15.36).

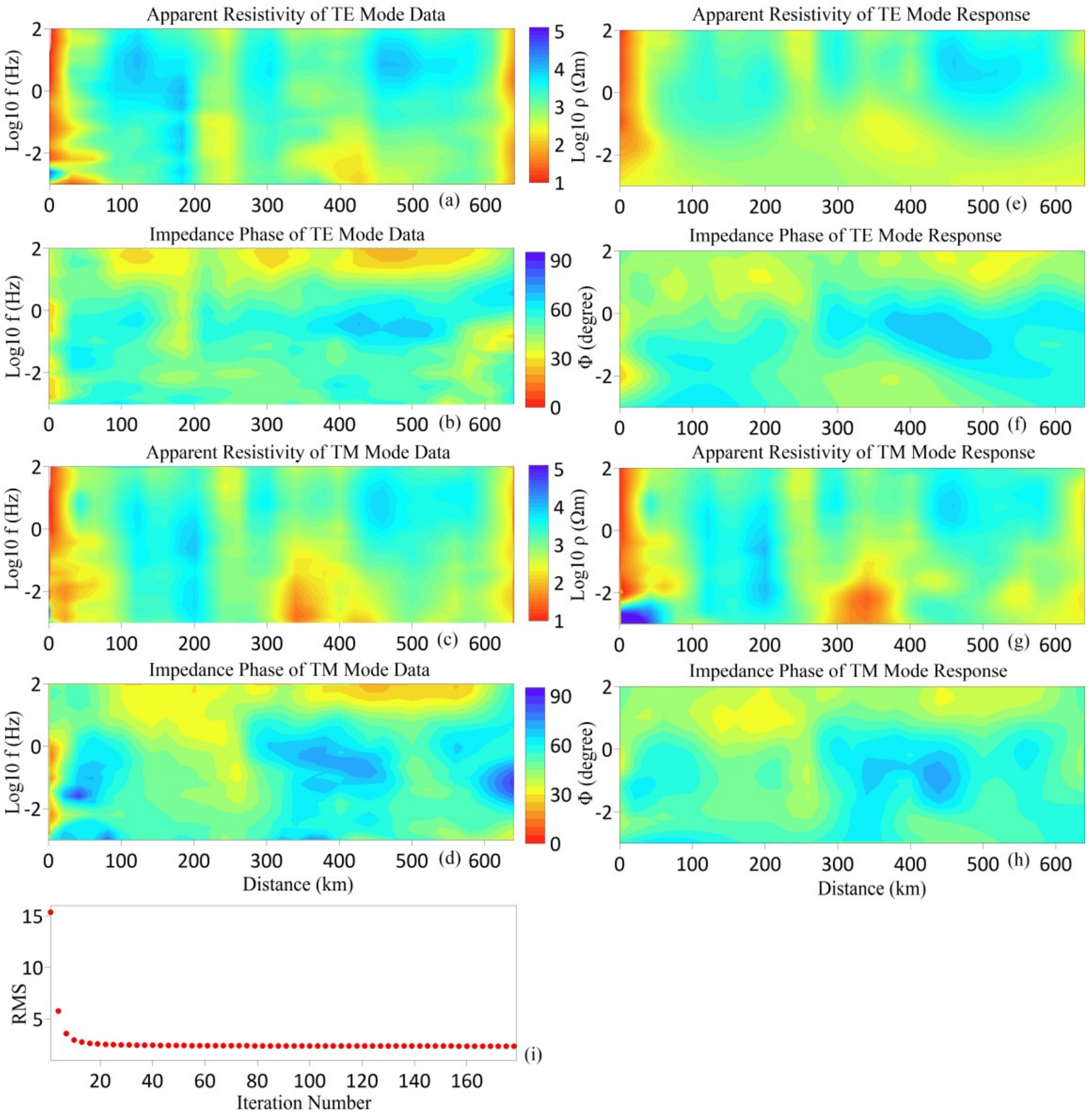

Figure S3. Comparison of the observed data and inversion responses located on line ZX (a-h), and normalized misfit for each iteration of the inversion (i). The preferred model is obtained after 91 iterations and fits data well with normalized misfit of 1.98 (from a starting model misfit of 14.47).

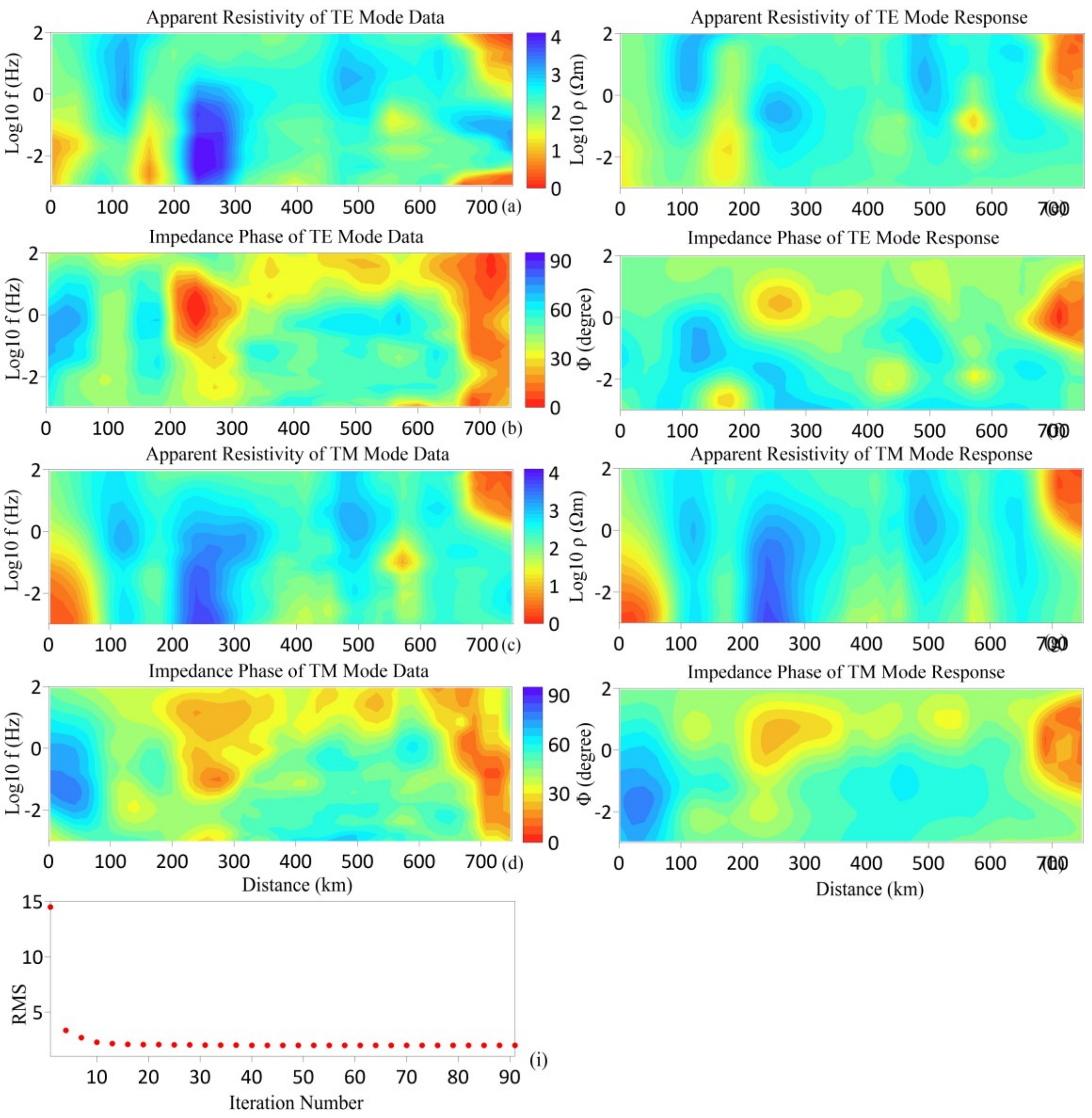

Figure S4a-d Maps of magnetotelluric phase tensors ellipses. Proposed tectonic boundaries in this study are shown in red dotted lines. Ellipses are plotted at the approximate depth of penetration (5 to 150 km), which depends on period. Please note that only evenly sparse stations are plotted for clear displaying. The NW-NE directed polarization distributed in different study area indicates different tectonic process and geodynamic background, respectively.

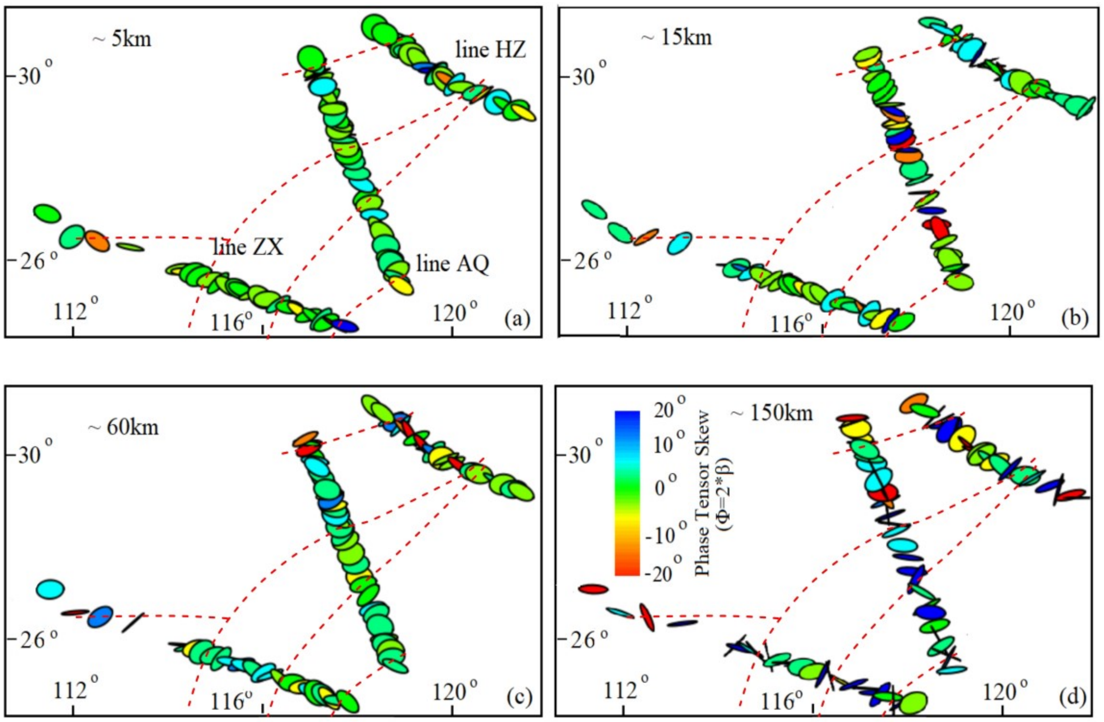

Figure S5. A demo of model tests. (a): original inversion model; (b): modified model; (c) test inversion model. The second inversion finds the same structure with preferred model to fit the data.

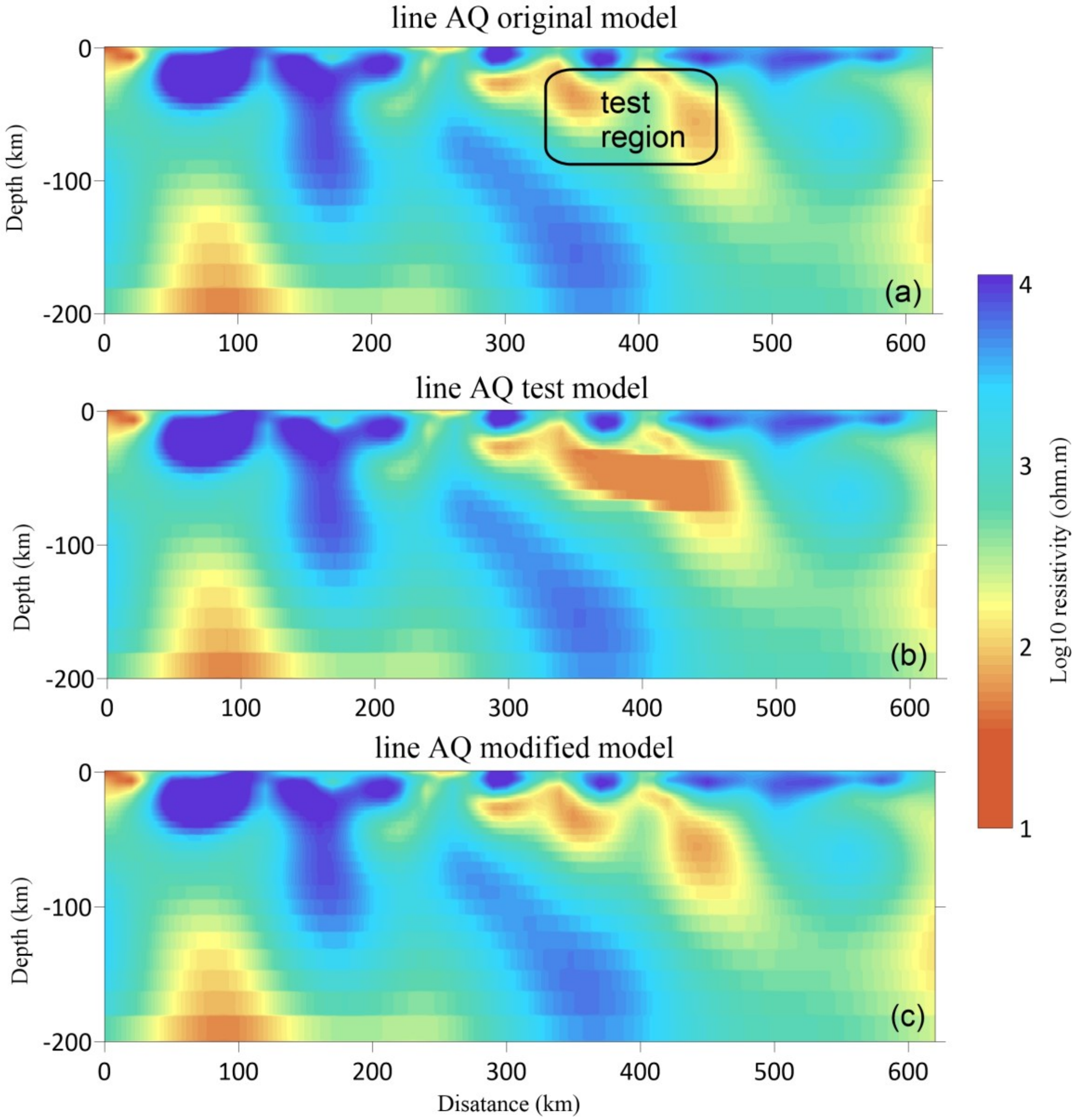

Figure S6. Typical geological and geophysical evidences for the tectonic evolution of South China. (a): heat-flow anomalies (data downloaded from http://chfdb.xyz/show.html); (b): Moho depth estimated by seismic data [1]; (c): Destiny difference of crust and upper mantle estimated by gravity data; (d): LAB depth estimated by seismic data [2]. The Neoproterozoic magmatism related to the continental accretion [3] are shown as stars in different color; monzonite gneiss, migmatite, and felsic metavolcanic rock samples [4] are shown in black spots; the major Cretaceous mafic rock belt [5] is shown in translucently green.

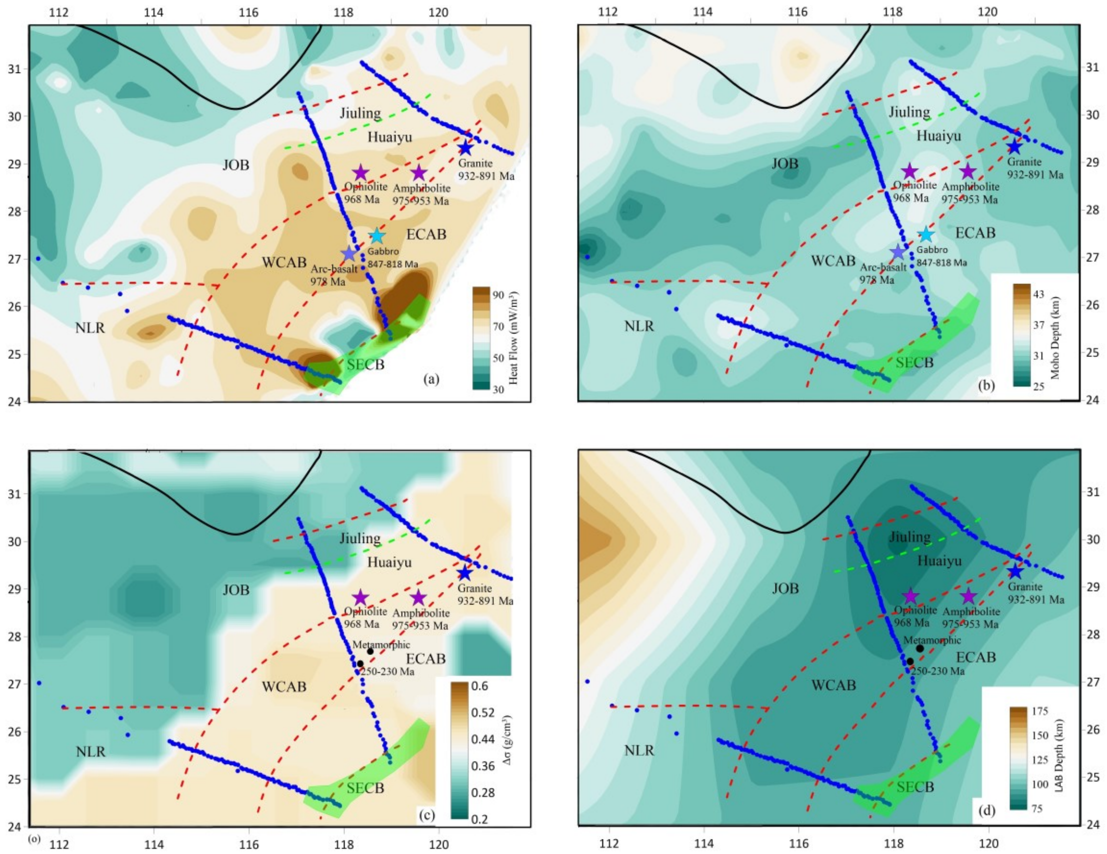

2. Supplementary References

[1] Feng Mei, An Meijian, Dong Shuwen, Tectonic history of the Ordos Block and Qinling Orogen inferred from crustal thickness. *Geophys. J. Int.* 210 (1), 303-320 (2017).

[2] An, M., Shi, Y. Lithospheric thickness of the Chinese continent. Phys. *Earth Planet. In.* 159, 257-266 (2006).

[3] Zhang, G. W., Guo, A. L., Wang, Y. J. , Li, S. Z., Dong, Y. P., Liu, S. F., Dong, Y. P., Liu, S. F., He, D. F., Cheng, S. Y., Lu, R. K., Yao, A. P. Tectonics of south china continent and its implications. *Science China Earth Sciences*. 56(11), 1804-1828 (2013).

[4] Lin, S. F., Xing, G. F., Davis, D. W., Yin, C. Q., Wu, M. L., Li, L. M., Jiang, Y., Chen, Z. H. Appalachian-style multi-terrane Wilson cycle model for the assembly of South China. *Geology*. https://doi.org/10.1130/G39806.1 (2018).

[5] Li, B., Jiang, S. Y., Zhang, Q., Zhao, H. X., Zhao, K. D. Cretaceous crust–mantle interaction and tectonic

evolution of Cathaysia Block in South China: Evidence from pulsed mafic rocks and related magmatism. *Tectonophysics.* 661, 136-155 (2015).
